# Supplementary material for: Added Value of Scintillating Element in Cerenkov-Induced Photodynamic Therapy
Source: Pharmaceuticals (Basel). 2023 Jan 18;16(2):143. doi: 10.3390/ph16020143 (PMC9963809; doi:10.3390/ph16020143)
Supplement: Supplementary file 1 [file pharmaceuticals-16-00143-s001.zip › pharmaceuticals-2119656-supplementary.pdf]

Supplementary Materials

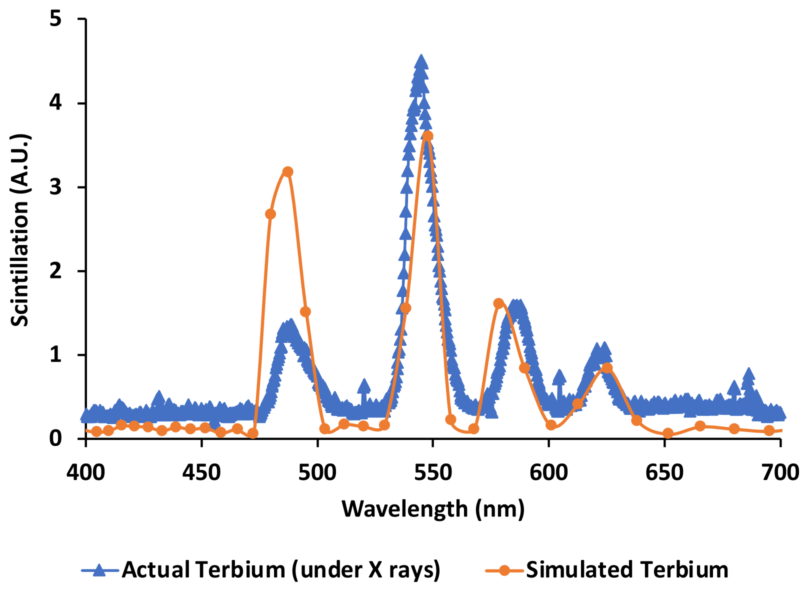

Figure S1. Comparison between Terbium (Tb) scintillation obtained after Gate simulations and after 320 kV X-ray exposure.

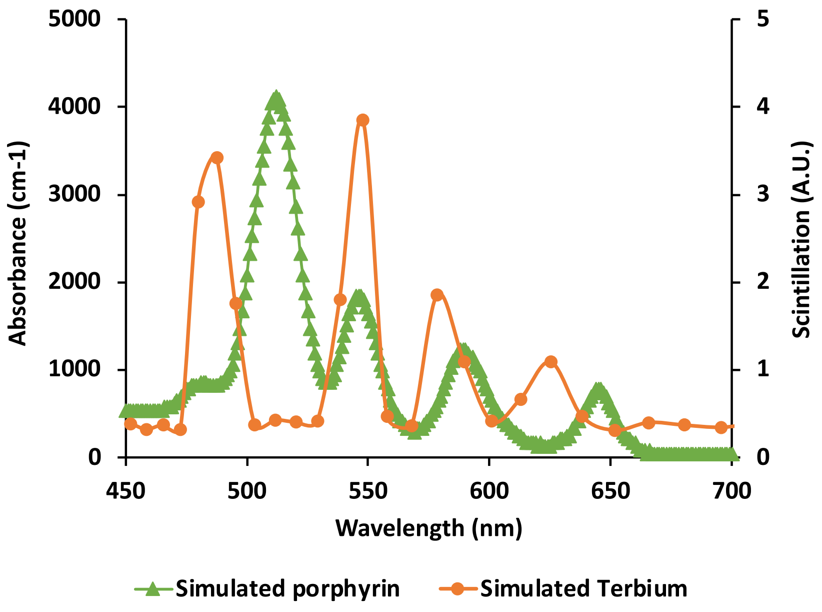

Figure S2. Spectrum overlap between simulated Terbium (Tb) scintillation and porphyrin (P1) Q bands.

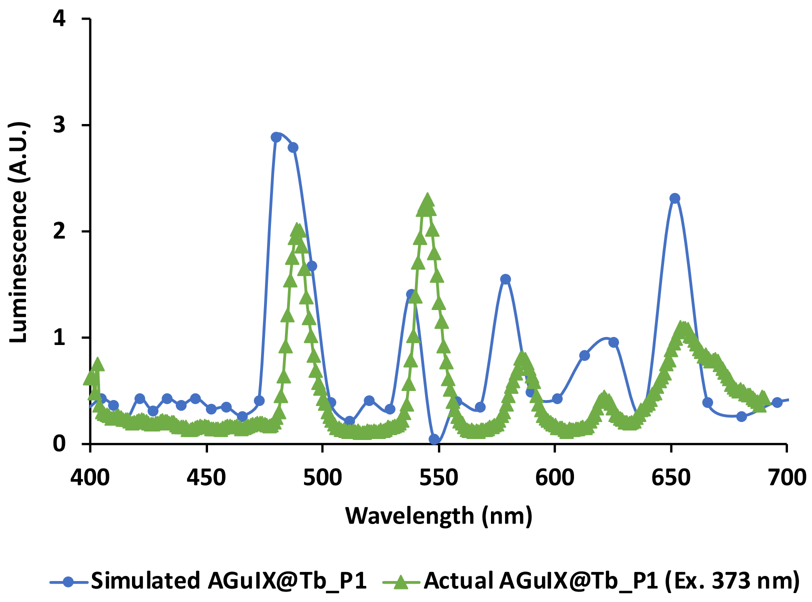

Figure S3. Comparison between AGuIX@Tb-P1 luminescence spectrum after actual UV excitation (373 nm) and the corresponding simulation.

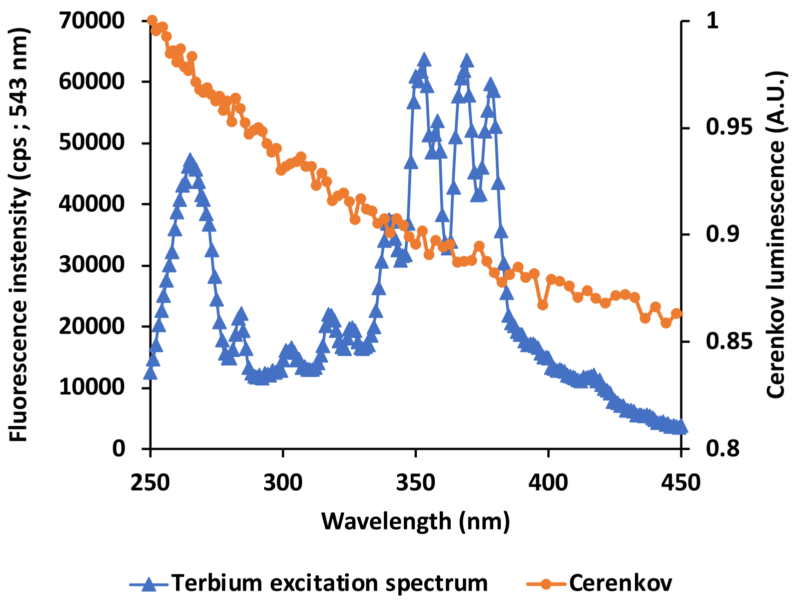

Figure S4. Terbium (Tb) excitation spectrum below 450 nm and Cerenkov typical spectrum overlap. To obtain excitation spectrum, we read the Tb emission at 543 nm after each excitation. The higher the fluorescence, the higher the absorption was.
